# Influence of Moderate Hyperbilirubinemia on Cardiorespiratory Control in Preterm Lambs

**DOI:** 10.3389/fphys.2019.00468

**Published:** 2019-04-26

**Authors:** Sally Al-Omar, Virginie Le Rolle, Nathalie Samson, Marie-Laure Specq, Melisande Bourgoin-Heck, Nathalie Costet, Guy Carrault, Jean-Paul Praud

**Affiliations:** ^1^Univ Rennes, Inserm, LTSI – UMR 1099, Rennes, France; ^2^Departments of Pediatrics and Physiology, Neonatal Cardiorespiratory Research Unit, University of Sherbrooke, Sherbrooke, QC, Canada; ^3^Univ Rennes, Inserm, EHESP, Irset (Institut de Recherche en Santé, Environnement et Travail), UMR_S 1085, Rennes, France

**Keywords:** hyperbilirubinemia, prematurity, heart rate variability, respiratory rate variability, cardiorespiratory interrelations

## Abstract

Hyperbilirubinemia (HB) is responsible for neonatal jaundice in 60% of term newborns and 90% of preterm infants. Neonatal HB can induce neurological damage (acute HB encephalopathy) and has been associated with persistent apneas. The objective of the present study was to investigate the immediate and delayed effects of moderate, clinically-relevant HB on cardiorespiratory control in preterm lambs. Two groups of five preterm lambs, namely control and HB, were studied. At day five of life, moderate HB (150–250 μmol/L) was induced and maintained during 17 h in the HB group while control lambs received a placebo solution. Six hours after HB onset, 7-h polysomnographic recordings with electrocardiogram (ECG) and respiratory (RESP) signals were performed to assess the immediate effects of HB on heart rate variability (HRV), respiratory rate variability (RRV), and cardiorespiratory interrelations. Identical recordings were repeated 72 h after HB induction to examine the delayed effects of HB on HRV, RRV and cardiorespiratory interrelations. Our results demonstrate a higher HRV and vagal activity immediately after induction of moderate HB. Meanwhile, a decrease in respiratory rate with an increase in both long- and short-term RRV was also noted, as well as a higher amplitude of the respiratory sinus arrhythmia and cardiorespiratory coupling. Seventy-two hours later, the alterations in HRV, RRV, and cardiorespiratory interrelations were attenuated, although a number of them were still present, suggesting a lasting influence of HB on the basal control of the cardiorespiratory system. Our results pave the way for studies in human preterms to assess the relevance of monitoring HRV, RRV, and cardiorespiratory interrelations to detect the acute neurological effects of HB and consequently adapt the treatment of neonatal jaundice.

## Introduction

Hyperbilirubinemia (HB) in newborns results from an imbalance between bilirubin production and its elimination. It is responsible for neonatal jaundice, which affects 60% of term newborns and 90% of preterm infants (Maisels and McDonagh, 2008; Watson, 2009). Preterm infants often develop a more severe and durable HB than term children (Watchko, 2016). Neonatal HB can induce acute and chronic neurological damage (Brites, 2012) and is the most common clinical condition requiring urgent treatment in the neonatal period (Kaplan et al., 2011).

When the binding capacity of albumin is exceeded in the blood, lipid-soluble unconjugated bilirubin can cross the blood-brain barrier and deposit in the central nervous system, mainly in the basal ganglia, the hippocampus, the subthalamic nuclei and the cerebellum (Diamond and Schmid, 1968; Bhutani and Johnson-Hamerman, 2015). At the cardiorespiratory level, several studies have linked HB to apnea of prematurity (Amin, 2004; DiFiore et al., 2004; Johnson et al., 2009; Amin et al., 2014), and apnea is considered a common clinical sign of acute bilirubin encephalopathy in preterm infants (Govaert et al., 2003; Gkoltsiou et al., 2008). In a recent study, we also showed a decrease in respiratory rate and an increase in apnea duration in preterm lambs with moderate clinically-relevant HB (150–250 μmol/L) (Specq et al., 2016).

A few studies have shown certain adverse effects of HB on autonomic function in term newborns (Uhrikova et al., 2015; Özdemir et al., 2017). For example, in 20 full-term icteric newborns (HB: 233–255 μmol/L), time domain and frequency domain analyses of heart rate variability (HRV) did not find any difference whereas non-linear indices detected an increased parasympathetic activity and/or decreased sympathetic activity (Uhrikova et al., 2015). Another study in 50 term neonates with severe HB showed some significant modifications in HRV indices (Özdemir et al., 2017). Overall, results indicate that HB can cause autonomic nervous system dysfunction in favor of parasympathetic predominance in neonates with neonatal jaundice. To our knowledge, there are no previous data on the effect of HB on autonomic activity in preterm newborns. In addition, there is no previous study of the effects of neonatal HB on respiratory rate variability (RRV) and cardiorespiratory interrelations.

The current study complements a previous publication on the same lambs showing electroencephalographic signs of acute HB encephalopathy and alterations of several cardiorespiratory reflex responses, as well as inflammation at the level of the cardiorespiratory centers (Specq et al., 2016). The latter led us to hypothesize that HB can also alter HRV, RRV, and cardiorespiratory interrelations in preterm lambs. Therefore, the objective of the present study was to investigate the immediate and delayed effects of a moderate and acute HB on HRV, RRV, and cardiorespiratory interrelations in preterm lambs.

## Materials and Methods

### Experimental Protocol

The experimental protocol has previously been described in detail (Specq et al., 2016) and was approved by the Ethics Committee for Animal Care and Experimentation of the University of Sherbrooke (protocol # 260–10). Experiments were conducted in 10 preterm lambs with a gestational age of 133 days (vs. 147 days at term). Preterm labor was induced by mifepristone injection (8 mg/kg), preceded by stimulation of lung maturation by betamethasone injection (12 mg × 2 mg) to the pregnant ewe (Boudaa et al., 2013; Nault et al., 2017). During the first 48 h of life, vital signs including heart rate, respiratory rate, transcutaneous oxygen saturation, rectal temperature, and blood glucose were continuously monitored. Experiments were started on the 5th day of life (D5) when the lambs had gained locomotor and feeding autonomy.

**FIGURE 1:**
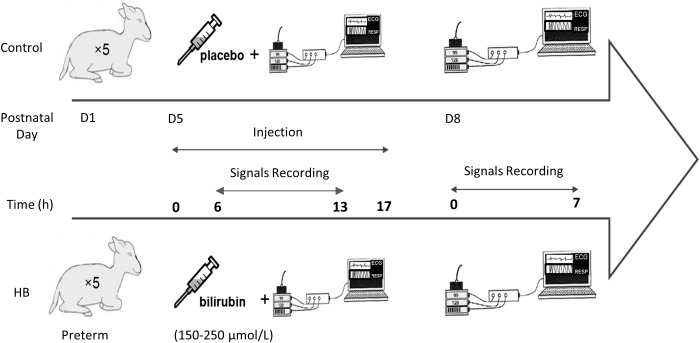
Experimental protocol.

Figure 1 describes the design of the study. Lambs were instrumented on D5. Instrumentation included electrodes for electrocardiogram (ECG) recording as well as abdominal and thoracic bands for recording respiratory thoraco-abdominal movements (RESP) by respiratory inductive plethysmography (Respitrace; NIMS Inc., Miami, FL, United States). Lambs were divided in a control group (*n* = 5) and a HB group (*n* = 5). Moderate HB was induced in the latter for 17 h *via* intravenous infusion of 20 mg/kg of bilirubin solution, stabilized with albumin and diluted in Ringer’s Lactate buffer. Control lambs received a placebo solution without bilirubin. Polysomnographic recordings were started 6 h after the beginning of the infusion, when bilirubinemia had stabilized at the fixed target values, between 150 and 250 μmol/L (Specq et al., 2016). To study the immediate effects of moderate HB on HRV, RRV and cardiorespiratory interrelations, ECG and RESP signals were continuously recorded during the 7-h nocturnal polysomnography (10:00 pm to 5:00 am). During these recordings, the non-sedated lambs could move freely in a ventilated Plexiglas chamber (1.2 m × 1.2 m × 1 m).

On D8, i.e., 72 h after the onset of HB induction, the same recordings were performed to assess the delayed effects of moderate HB on cardiorespiratory control.

### A Dedicated Signal Processing Framework

**FIGURE 2:**
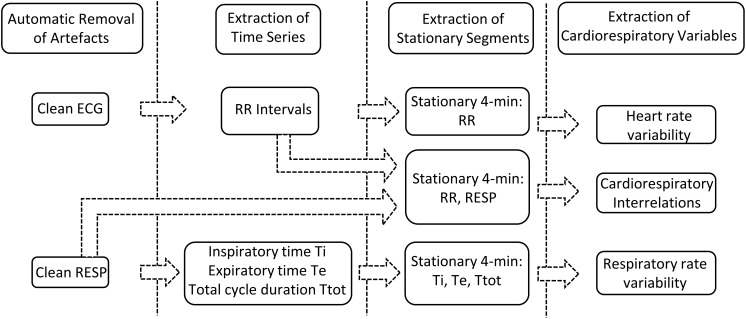
Signal processing approach.

Our previously developed semi-automated processing approach was applied on ECG and RESP signals recorded from non-sedated animals. Each step of the developed approach, represented in Figure 2, has been detailed previously (Al-Omar et al., 2018) and is briefly presented hereafter.

First, movement artifacts were automatically removed from RESP signals based on the K-nearest-neighbors classifier (Manocha and Girolami, 2007) applied on a 1-min sliding window with 50% overlap. On each 1-min window the following features were estimated (the absolute difference between the maximum and minimum values of the segment, the mean of the segment divided by its maximum value, the standard deviation, the kurtosis, the root-mean squared value and the power spectral density in the frequency bands 0.02–0.4 and 0.4–2 Hz ). Each 1-min segment was hence classified into a clean or artifacted segment. Clean RESP signals were subsequently filtered using a simple bandpass filter to remove both baseline and high frequency noise. In addition, in order to reduce cardiac artifacts, a Savitzky–Golay filter was applied to smooth the RESP signal without distortion. Following extraction of clean RESP segments, simultaneous ECG segments were retained. A pre-processing step to clean ECG segments from additional motion-induced artifacts was applied (Strasser et al., 2012), then a low-pass filter was used to reduce noise.

Secondly, the following time series were extracted from the remaining clean segments of duration equal or greater than 4 min: RR intervals from the ECG signal; expiratory time Te, inspiratory time Ti and total breathing cycle time Ttot from the RESP signal. RR intervals were automatically extracted by detecting each QRS complex as the maximum above a manually-fixed threshold, followed by a systematic visual verification and manual correction when necessary. Te and Ti were computed as the time elapsed between successive maxima and minima or between successive minima and maxima of the RESP signal, respectively, and Ttot as the time duration between two successive minima of the RESP signal (Navarro et al., 2015).

Thirdly, stationary segments were obtained by applying a specific stationarity test on the extracted time series (Borgnat et al., 2010). The test considered stationarity as time invariance in the time-frequency spectrum. It was applied on a 4-min sliding window with 50% overlap in order to extract the maximum number of 4-min stationary segments. Stationary segments of the RESP signal were, similarly, obtained for analysis of the cardiorespiratory interrelations.

### Data Analysis

All analyses were applied on the 4-min RR, Te, Ti, Ttot and RESP stationary segments obtained as explained above.

#### Heart Rate Variability

As previously detailed (Al-Omar et al., 2018), linear HRV analysis included measurements in both the time-domain [mean; standard deviation, SD; coefficient of variation (%) = ratio of SD to the mean (Harrington et al., 2003); mean squared differences of successive RR intervals, RMSSD; skewness and kurtosis] and the frequency domain [power in the low-frequency (LF, 0.02–0.25 Hz) and high-frequency (HF, 0.25–2 Hz) spectral bands, total power, LF/HF ratio]. Nonlinear analyses included SD1 and SD2 from the Poincaré plot, sample entropy (SampEn), detrended fluctuation analysis (alpha1) and deceleration (DC) and acceleration (AC) capacities (Campana et al., 2010; Pan et al., 2016).

#### Respiratory Rate Variability

Linear analyses performed on Te, Ti and Ttot time series included the mean, SD, the coefficient of variation, as well as skewness and kurtosis. Nonlinear analyses were also performed to compute SD1 and SD2 of the Poincaré plot and the SampEn (Beuchée et al., 2012).

#### Cardiorespiratory Interrelations

Cardiorespiratory interrelation analyses have previously been detailed (Al-Omar et al., 2018). Interrelations were evaluated by extracting (i) the Pearson (r^2^) and the non-linear (h^2^) correlation coefficients; (ii) the maximum magnitude squared coherence; (iii) the mean phase coherence γ_*RR,RESP*_ as a phase synchronization index; (iv) the cardioventilatory coupling and (v) the magnitude of the respiratory sinus arrhythmia (RSA). The latter was computed as the difference between the maximum and the minimum RR in a respiratory cycle (Van Roon, 1998). In addition, the mean number of heartbeats occurring in each expiration (MeanRRExpi) or inspiration (MeanRRInspi) was extracted.

### Statistical Analysis

The normality of the distribution of all studied variables was assessed using the Shapiro-Wilk test. When normality was rejected, a Box-Cox transformation with λ = 0 or λ = 0.5 was applied (log10 or square root transformations). Results were expressed as median (quartile 1; quartile 3).

In order to test the immediate effects of HB on each variable, we compared the control and HB groups on D5 using a mixed ANOVA for repeated measures (Cnaan et al., 1997), as several non-independent measurements were performed in each lamb. In the mixed ANOVA model, the lamb was considered as a random effect and the group (HB vs. control) as a fixed effect. The same analysis was performed on D8 in order to assess the delayed effects of HB. Finally, in order to test whether the differences between groups varied from D5 to D8, we performed a global analysis using an ANOVA mixed model with the lamb as a random effect, and the time (D5 vs. D8), the group (HB vs. control) and their interaction as fixed effects. The statistical significance of the interaction term indicated whether differences between groups varied across time. For all ANOVA tests, *p*-values <0.05 were considered as statistically significant. However, since the number of newborn lambs was relatively small (due to the high complexity of the preterm ovine model as well as ethical constraints), potential trends were also considered when *p*-values <0.1.

Statistical analyses were performed using the “nlme” R software package (Pinheiro et al., 2017).

## Results

**FIGURE 3:**
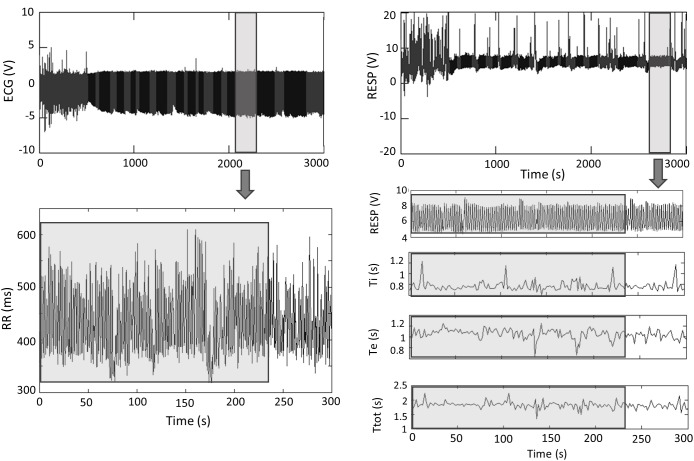
ECG and RESP signals with the corresponding extracted times series. Analyses were performed on 4-min stationary segments. Ti, inspiratory time; Te, expiratory time; Ttot, total respiratory cycle duration. Gray, artifacts; Black, clean.

Figure 3 represents an example of ECG and RESP signals with the corresponding extracted time series.

### Immediate Effects of Moderate Hyperbilirubinemia on Basal Cardiorespiratory Control

#### Heart Rate Variability

A mean of 9 (±5) and 7 (±4) stationary RR 4-min segments per lamb were obtained in control and HB groups, respectively. Table 1 presents the significant results obtained for HRV analysis. For the linear analyses, global variability represented by the coefficient of variation in the time domain, as well as HF, LF, and total power in the frequency domain, tended to be higher in HB lambs compared to controls. In addition, nonlinear analyses yielded a significantly higher deceleration and acceleration capacities in HB lambs. All other analyses gave *p*-values between 0.17 and 0.58.

**Table 1:** Immediate effects of hyperbilirubinemia on heart rate variability.

HRV Time domain	median (Q1;Q3) Control	median (Q1;Q3) HB	Mixed ANOVA *p*-value
Coeff. of variation (%)	4.5 (2.6;5.8)	4.8 (4.2;6.5)	0.1^∗^
**Frequency domain**			
HF (ms^2^)	7.3 (3.4;16)	19.6 (14.3;32.8)	0.07^∗^
LF (ms^2^)	28 (14.4;54.9)	53.3 (35.5;70.3)	0.06^∗^
Total power (ms^2^)	43.5 (18.7;71.2)	76.8 (49.2;109.4)	0.1^∗^
**Nonlinear analyses**			
DC (ms)	0.2 (0.04;0.5)	1.2 (0.8;1.7)	0.02^∗∗^
AC (ms)	0.1 (0.01;0.3)	0.7 (0.6;1.05)	0.005^∗∗^

*HRV, heart rate variability; Q1 and Q3, quartile 1 and 3; HB, hyperbilirubinemic lambs; HF, power in the high frequency band; LF, power in the low frequency band; DC, deceleration capacity; AC, acceleration capacity. ^∗^0.05 < p ≤ 0.1; ^∗∗^p < 0.05, hyperbilirubinemia vs. control lambs. Results with p > 0.1 are not shown.*

#### Respiratory Rate Variability

Between 4 and 7 stationary RR 4-min segments per lamb were obtained on average for Ttot, Ti and Te in the control and HB groups. Table 2 presents the significant results obtained in HB lambs compared to controls. First, respiratory rate was significantly lower in HB lambs. For the Ttot series, the mean and standard deviation, as well as the SD1 and SD2 indices of the Poincaré plot were significantly higher in HB lambs. The same variables extracted from the Ti series were significantly higher, whereas SampEn tended to be lower in HB lambs. For the Te series, the mean and standard deviation as well as the SD1 and SD2 indices were higher in HB lambs. All other analyses gave *p*-values between 0.23 and 0.96.

**Table 2:** Immediate effects of hyperbilirubinemia on respiratory rate variability.

RRV Variables	median (Q1;Q3) Control	median (Q1;Q3) HB	Mixed ANOVA *p*-value
**Ttot**			
Mean (s)	0.8 (0.7;0.8)	1.5 (1.2;1.7)	0.001^∗∗^
SD (s)	0.1 (0.08;0.2)	0.2 (0.1;0.3)	0.006^∗∗^
SD1 (s)	0.08 (0.06;0.1)	0.2 (0.1;0.2)	0.006^∗∗^
SD2 (s)	0.1 (0.1;0.2)	0.3 (0.1;0.4)	0.01^∗∗^
**Ti**			
Mean (s)	0.4 (0.3;0.4)	0.7 (0.6;0.8)	0.002^∗∗^
SD (s)	0.05 (0.03;0.06)	0.1 (0.07;0.1)	0.002^∗∗^
SD1 (s)	0.04 (0.03;0.05)	0.09 (0.06;0.1)	0.001^∗∗^
SD2 (s)	0.05 (0.04;0.07)	0.1 (0.07;0.2)	0.003^∗∗^
SampEn	4.1 (3.7;4.4)	3.3 (3.1;3.5)	0.07^∗^
**Te**			
Mean (s)	0.4 (0.4;0.5)	0.8 (0.7;0.9)	0.004^∗∗^
SD (s)	0.09 (0.06;0.1)	0.2 (0.1;0.2)	0.02^∗∗^
SD1 (s)	0.07 (0.05;0.08)	0.1 (0.07;0.1)	0.08^∗^
SD2 (s)	0.09 (0.07;0.1)	0.2 (0.1;0.2)	0.02^∗∗^

*RRV, respiratory rate variability; Ttot, total duration of the breathing cycle; SD, standard deviation; SD1 and SD2, indices of short-term and long-term variability, respectively, of the Poincaré plot; Ti, inspiratory time duration; SampEn, sample entropy; Te, expiratory time duration. See Table 1 for other abbreviations.*

#### Cardiorespiratory Interrelations

Cardiorespiratory interrelations were analyzed on a mean of 5 (±2) and 8 (±6) stationary RR and RESP 4-min segments in control and HB groups, respectively. Table 3 presents the significant differences in HB lambs compared to controls. The synchronization index (mean phase coherence γ_RR,RESP_), the number of RR intervals in expiration and inspiration and the RSA amplitude were significantly higher in HB lambs. All other analyses gave *p*-values between 0.18 and 0.72.

**Table 3:** Immediate effects of hyperbilirubinemia on cardiorespiratory interrelations.

Cardiorespiratory interrelations	median (Q1;Q3) Control	median (Q1;Q3) HB	Mixed ANOVA *p*-value
RSA (ms)	7.5 (3.5;8.1)	15.7 (12.2;19.4)	0.07^∗^
γ_*RR,RESP*_ (n.u.)	0.01 (0.008;0.03)	0.1 (0.09;0.1)	0.003^∗∗^
MeanRR_*Expi*_ (n.u.)	1.5 (1.3;1.7)	2.4 (2.2;2.7)	<0.001^∗∗^
MeanRR_*Inspi*_ (n.u.)	1.2 (1.1;1.4)	2.08 (1.9;2.3)	<0.001^∗∗^

*RSA, respiratory sinus arrhythmia amplitude; γ_*RR,RESP*_, synchronization index; MeanRR_Expi_ and MeanRR_Inspi_, mean number of heartbeats occurring in each expiration and inspiration, respectively; n.u., no units. See Table 1 for other abbreviations.*

### Delayed Effects of Moderate Hyperbilirubinemia on Basal Cardiorespiratory Control

#### Heart Rate Variability

A mean of 9 (±6) and 11 (±7) stationary RR 4-min segments per lamb were obtained in control and HB groups, respectively. As presented in Table 4, only the deceleration and acceleration capacities were significantly higher in the HB vs. control lambs on D8. All other analyses gave *p*-values between 0.19 and 0.98.

**Table 4:** Delayed effects of hyperbilirubinemia on heart rate variability.

HRV Nonlinear analysis	median (Q1;Q3) Control	median (Q1;Q3) HB	Mixed ANOVA *p*-value
DC (ms)	0.3 (0.1;0.5)	0.5 (0.2;1.04)	0.005^∗∗^
AC (ms)	0.1 (−0.02;0.2)	0.2 (0.09;0.6)	0.02^∗∗^

*See Table 1 for the definition of abbreviations.*

In addition, the differences between HB and control lambs were greater on D5 vs. D8 for HF (*p* = 0.006), total power (*p* = 0.09), and DC (*p* = 0.1).

#### Respiratory Rate Variability

Between 5 and 9 stationary 4-min segments per lamb were obtained for Ttot, Ti and Te in both the control and HB groups. Significant differences in HB vs. control lambs are presented in Table 5. On D8, the mean and standard deviation of Ttot tended to be higher in HB lambs, and the SD1 index was significantly higher vs. control lambs. Also, for the Te time series, the mean, standard deviation, and the SD1 and SD2 indices were significantly higher in HB lambs, while the skewness was lower. No significant differences between HB and control lambs were observed for the Ti time series. All other analyses gave *p*-values between 0.17 and 0.89.

**Table 5:** Delayed effects of hyperbilirubinemia on respiratory rate variability.

RRV Variables	median (Q1;Q3) Control	median (Q1;Q3) HB	Mixed ANOVA *p*-value
**Ttot**			
Mean (s)	0.9 (1;1.1)	1.9 (1.04;1.7)	0.06^∗^
SD (s)	0.1 (0.1;0.2)	0.2 (0.1;0.3)	0.1^∗^
SD1 (s)	0.1 (0.1;0.1)	0.1 (0.1;0.2)	0.03^∗∗^
**Te**			
Mean (s)	0.6 (0.5;0.6)	0.8 (0.6;1)	0.006^∗∗^
SD (s)	0.09 (0.06;0.1)	0.1 (0.1;0.2)	0.002^∗∗^
Skewness	0.3 (0.02;0.9)	−0.3 (−0.7;0.6)	0.004^∗∗^
SD1 (s)	0.07 (0.05;0.1)	0.1 (0.08;0.1)	0.02^∗∗^
SD2 (s)	0.1 (0.07;0.2)	0.2 (0.1;0.2)	0.02^∗∗^

*See Tables 1, 2 for the definition of abbreviations.*

In addition, the differences between HB and control lambs for the mean (*p* < 0.0001) and SD1 (*p* = 0.1) of Ttot, the mean (*p* < 0.0001), SD1 (*p* = 0.04) and Sampen (*p* = 0.06) of Ti, and the mean (*p* = 0.005) of Te were decreased on D8 compared to D5.

#### Cardiorespiratory Interrelations

Cardiorespiratory interrelations were analyzed on a mean of 8 (±2) and 9 (±5) stationary RR and RESP 4-min segments in control and HB lambs, respectively. As shown in Table 6, the number of RR intervals during an expiration and an inspiration were significantly higher in the HB vs. control lambs, while the synchronization index tended to be higher. All other analyses gave *p*-values between 0.37 and 0.99.

**Table 6:** Delayed effects of hyperbilirubinemia on cardiorespiratory interrelations.

Cardiorespiratory interrelations	median (Q1;Q3) Control	median (Q1;Q3) HB	Mixed ANOVA *p*-value
γ_*RR,RESP*_ (n.u.)	0.04 (0.02;0.08)	0.1 (0.05;0.2)	0.09^∗^
MeanRR_*Expi*_ (n.u.)	1.9 (1.7;2.4)	2.5 (2.3;2.8)	0.04^∗∗^
MeanRR_*Inspi*_ (n.u.)	1.5 (1.4;1.7)	1.9 (1.6;2.1)	0.02^∗∗^

*See Table 3 for the definition of abbreviations.*

In addition, the differences between HB and control lambs for the number of RR intervals during an expiration (*p* = 0.0001) or an inspiration (*p* < 0.0001), the RSA amplitude (*p* = 0.1) and the synchronization index (*p* = 0.0001) were decreased on D8 compared to D5.

## Discussion

Our study reports unique results on the immediate and delayed effects of sustained moderate HB on the basal cardiorespiratory control of preterm lambs. Compared to control lambs, we observed a number of alterations of basal cardiorespiratory control in HB lambs, including: (1) a generally higher global HRV on D5 and a higher parasympathetic activity on both D5 and D8; (2) a lower respiratory rate accompanied by a higher variability of Ttot on both D5 and D8; (3) a higher duration of inspiration with a higher long- and short-term variability and a lower complexity on D5 only; (4) a higher duration of expiration with a higher variability on both D5 and D8; (5) a higher amplitude of the RSA on D5, as well as a higher synchronization index and number of RR intervals during expiration and inspiration on both D5 and D8. In addition, further statistical analysis comparing the delayed (D8) vs. the immediate (D5) effects of HB on HRV showed that the parasympathetic activity significantly decreased from D5 to D8. Similarly, alterations of the indices of RRV and cardiorespiratory interrelations generally decreased from D5 to D8.

Bilirubin is a powerful antioxidant at physiological levels but can become neurotoxic at abnormally high levels, especially in preterm infants. However, the effects of moderate clinically-relevant HB on the autonomic nervous system function still remain far from being completely understood in preterm infants. As indicated earlier, the study presented herein is part of a larger study conducted by our team on the effect of HB in preterm lambs. Two previous publications in these preterm lambs have highlighted that HB is responsible for: (i) abnormal swallowing-breathing coordination during bottle-feeding (Bourgoin-Heck et al., 2015); (ii) abnormal cardiorespiratory responses to stimulation of laryngeal mucosal chemoreceptors and bronchopulmonary sensory C fibers, as well as to hypoxic exposure. These findings were accompanied by an inflammation in the dorsal vagal complex region, recognized as an important component of brainstem cardiorespiratory centers (Specq et al., 2016). Finally, the electroencephalogram showed a bilirubin encephalopathy with a monotonous electrical activity, which was no longer modulated by the states of consciousness (Specq et al., 2016).

### Immediate Effects of Moderate Hyperbilirubinemia on Basal Cardiorespiratory Control

#### Heart Rate Variability

Our observation of a significant increase in total power (index of global autonomic activity), spectral power of HF (index of parasympathetic activity) and LF (index of both parasympathetic activity and sympathetic activity) oscillations of RR intervals in HB lambs suggests that moderate HB increases parasympathetic activity in preterm lambs (Valenza et al., 2018). This is further supported by the increase in DC and AC capacities (Campana et al., 2010; Pan et al., 2016).

Two studies have previously analyzed the effects of HB on the autonomic nervous system. A slight increase in parasympathetic activity was associated with HB in 20 term neonates (mean bilirubinemia: 249.5 μmol/L), with a shift to sympathetic activation during phototherapy (Uhrikova et al., 2015). Similarly, a predominance of parasympathetic activity was observed in 50 term neonates with severe HB (376 ± 94 μmol/L) receiving phototherapy compared to 50 healthy term newborns (Özdemir et al., 2017). Overall, our results further show that similar conclusions can be drawn in preterm subjects with a milder HB.

#### Respiratory Rate Variability

To our knowledge, there are no literature data on the effects of HB on RRV in preterm infants. Overall, our results show that a moderate HB alters RRV by increasing (i) the duration of both inspiration and expiration, (ii) the variability in Ttot, Ti and Te series, as well as (iii) the complexity of the Ti series. Interestingly, a prospective observational study in preterm infants (gestational age: 27–33 weeks) has shown that the presence of unconjugated HB is associated with central apneas in preterm infants, persisting after normalization of bilirubinemia (Amin and Wang, 2015).

#### Cardiorespiratory Interrelations

No previous study to our knowledge has assessed the effects of HB on cardiorespiratory interrelations. Our present results show that RSA tends to increase in the HB group. Central mechanisms contributing to the generation of RSA include direct modulation of central cardiac vagal outflow by the central respiratory drive (Louw et al., 2008), such that an increase in RSA amplitude is generally considered as indicating an increased parasympathetic tone (Carroll et al., 2012; Elstad et al., 2018). This is consistent with the increase in HF, DC, and AC reported above. In addition, an increase in cardiorespiratory synchronization was observed in the HB group. The physiological mechanisms responsible for cardiorespiratory synchronization are unclear (Lucchini et al., 2017). Some authors have suggested that the latter is related to a central neural regulation mechanism (Schäfer et al., 1999) with a high level of parasympathetic activity (Jerath et al., 2014). Finally, the increase in the number of RR intervals in expiration and inspiration can be explained by the prolongation of inspiratory and expiratory durations with a slight, non-significant increase in RR [mean RR (SD) = 306 (56) in HB vs. 276 (27) in control lambs on D5, *p*-value = 0.4].

### Delayed Effects of Moderate Hyperbilirubinemia on Basal Cardiorespiratory Control

Our results show that certain alterations in HRV, RRV, and cardiorespiratory interrelation indices persisted 72 h after HB, although they were generally attenuated. In particular, the parasympathetic activity decreased from D5 to D8 in HB lambs, but was still higher than in control lambs on D8. The persistence of alterations in basal cardiorespiratory control is likely due, at least in part, to the persisting inflammation of the dorsal vagal complex region previously observed 72 h after HB in preterm lambs (Specq et al., 2016). Taken together, the present results combined with our previous findings show that moderate HB in preterm lambs alters both the basal cardiorespiratory control drive and the cardiorespiratory reflex responses to various stimuli for at least a few days (Specq et al., 2016). This is also in agreement with a previous study in rats showing the presence of bilirubin deposits on the ventral medullary surface after induction of a severe and transient HB (425 μmoles/L for ∼2 h), together with a decreased hypoxic ventilatory response (Mesner et al., 2008). Moreover, the bilirubin encephalopathy observed via EEG recording in our previous study could mitigate the cortical neuronal influences on the brainstem cardiorespiratory centers, contributing in turn to the decrease in respiratory rate and Ti complexity, as well as to the increase in cardiorespiratory synchronization.

### Study Limitations

The first limitation is related to the small number of lambs, which is due to the very high cost of preterm lambs, the complexity of post-natal continuous care and a 25% mortality rate. Of note, our team is currently the only group, to our knowledge, to use a chronic model of preterm lamb (Samson et al., 2018). In addition, the absence of sedation, which is crucial to avoid its adverse effects on cardiovascular and respiratory function, yielded ECG and RESP signals with many movement artifacts and rare stationary segments. Finally, the use of non-calibrated respiratory inductive plethysmography excluded the analysis of tidal volume variability, which may have yielded additional information. The choice of the use of such minimally invasive instrumentation was nevertheless deliberate to allow studying non-sedated animals.

## Conclusion

The present results obtained in preterm lambs suggest that a moderate and sustained HB, as encountered daily in neonatal care units, is responsible for alterations in basal cardiorespiratory control in preterm newborns. Importantly, there is currently no reliable means to ascertain the level of bilirubin that can cause cerebral toxicity in a given preterm newborn. We propose that the continuous monitoring of HRV, RRV, and cardiorespiratory interrelations may be one of the various ways used to recognize that a toxic level of HB has been reached, leading in turn to consider a more rapid treatment and obtain a better long-term prognosis. This suggestion, however, must be supported by further experiments in preterm human infants.

## Ethics Statement

This study was carried out in accordance with the recommendations of the Ethics Committee for Animal Care and Experimentation of Sherbrooke University (protocol # 260–10).

## Author Contributions

NS, M-LS, MB-H, and J-PP conceived and designed the experimental protocol of the study. ML-S, MB-H, and NS performed the animal experiments. SA-O, VL, and GC designed, optimized, and adapted the signal processing chain. SA-O and NC analyzed the data. SA-O, VL, GC, NC, and J-PP interpreted the results. SA-O prepared the figures and drafted the manuscript. All the authors revised, read and approved the final version of the manuscript.

## Conflict of Interest Statement

The authors declare that the research was conducted in the absence of any commercial or financial relationships that could be construed as a potential conflict of interest.
